# Morphological Computation in Plant Seeds for a New Generation of Self-Burial and Flying Soft Robots

**DOI:** 10.3389/frobt.2021.797556

**Published:** 2021-11-26

**Authors:** Barbara Mazzolai, Stefano Mariani, Marilena Ronzan, Luca Cecchini, Isabella Fiorello, Kliton Cikalleshi, Laura Margheri

**Affiliations:** Bioinspired Soft Robotics Laboratory, Istituto Italiano di Tecnologia, Genova, Italy

**Keywords:** bioinspired robotics, soft robotics, embodied intelligence, plant biology, smart materials, plant biomechanics, seeds dispersal

## Abstract

Plants have evolved different mechanisms to disperse from parent plants and improve germination to sustain their survival. The study of seed dispersal mechanisms, with the related structural and functional characteristics, is an active research topic for ecology, plant diversity, climate change, as well as for its relevance for material science and engineering. The natural mechanisms of seed dispersal show a rich source of robust, highly adaptive, mass and energy efficient mechanisms for optimized passive flying, landing, crawling and drilling. The secret of seeds mobility is embodied in the structural features and anatomical characteristics of their tissues, which are designed to be selectively responsive to changes in the environmental conditions, and which make seeds one of the most fascinating examples of morphological computation in Nature. Particularly clever for their spatial mobility performance, are those seeds that use their morphology and structural characteristics to be carried by the wind and dispersed over great distances (i.e. “winged” and “parachute” seeds), and seeds able to move and penetrate in soil with a self-burial mechanism driven by their hygromorphic properties and morphological features. By looking at their motion mechanisms, new design principles can be extracted and used as inspiration for smart artificial systems endowed with embodied intelligence. This mini-review systematically collects, for the first time together, the morphological, structural, biomechanical and aerodynamic information from selected plant seeds relevant to take inspiration for engineering design of soft robots, and discusses potential future developments in the field across material science, plant biology, robotics and embodied intelligence.

## Introduction

Seed dispersal is an interesting area for ecology, plant diversity and adaptation, and climate change (Traveset et al., 2014; Johnson et al., 2019). More recently, seed functioning and dispersion abilities have attracted the interest also of material scientists and engineers (Elbaum et al., 2007; Armon et al., 2011; Yao and Ishii, 2019; Geer et al., 2020). The strategies of seed dispersal are in fact characterized by an interesting mass and energy efficiency, adaptability, robustness and the embodiment of remarkable forms of morphological intelligence (Pandolfi and Izzo, 2013).

Seeds transport is “passive”, meaning that lack active metabolism and no internal energy is produced. The movement is instead powered by the intrinsic material and structural features of the seed tissues (Fratzl and Barth, 2009; Abraham, 2018; Abraham and Elbaum, 2013), which endow them with high responsiveness to changing environmental conditions (e.g., humidity and temperature), or the ability to exploit environmental factors as mobility vectors (wind, water, animals). In this sense, seeds represent one of the most interesting and significant examples of morphological computation in Nature, providing a wide collection of physical and mechanical features optimized for system passive flying, landing, crawling and drilling.

By looking at the natural dispersion strategies of seeds, new design principles can be extracted and used as inspiration for artificial systems able to interact with the surrounding environment by exploiting their morphological computation abilities.

With this in mind, this mini-review systematically reports, for the first time together, the morphological, structural, biomechanical and aerodynamic information from selected “diaspores”, including both seeds and fruits - and hereafter called “seeds” for the sake of simplicity.

Several remarkable characteristics of seed morphology and structure are involved in their dispersal strategy, such as the adhesive surfaces on burdock seeds that inspired the invention of the most famous hook and loop mechanical interlocker, VELCRO^®^ (De Mestral, 1961). From an engineering design of dynamic solutions point of view, this study focuses on those seeds whose self-burial and flying abilities allow an efficient 3-D movement in space, making them relevant examples to take inspiration from for innovative soft robots. Investigation methodologies and tools used for the biological systems analysis and for the definition of the data are also described.

## Self-Burying Seeds

One mode of seed dispersal is through explosive or wind lift followed by self-burial thanks to sterile appendages capable of hygroscopic movements allowing the seeds to move across and into the soil (Evangelista et al., 2011). This is the case for the seeds of the Geraniaceae family, which includes 841 species and 7 genera of annual and perennial herbs (Hutchinson, 1969; The Plant List, 2013).

### Morphology and Structure

Geraniaceae fruits generally have a five-carpelled schizocarp and a central axis called *columella*. Each carpel has a mericarp structure, which contains the seed, and a sterile robust tissue called awn, that extends along the central axis (Yeo, 1984). Differentiation in the family on the seed dispersal mode has led to a seed dispersal classification: the “Erodium-type” (ET), the “carpel-projection type” (CP), the “seed-ejection type” (SE), and the “inoperative type” (IT) (Marcussen and MeseguerYeo, 1984; 2017).

Erodium-type (ET) discharge, called after the genus *Erodium*, is characterized by an explosive launch in the air, in dry conditions, of the awn and mericarp fused. After detachment from the *columella*, the awn rapidly coils to its natural form releasing the mechanical energy previously stored. Once on the ground the awn can coil and uncoil in response to humidity changes and facilitating its self-burial (Yeo, 1984; Jung et al., 2014).

ET discharge was suggested to be the most primitive dispersal mode, from which the other types originated (Marcussen and Meseguer, 2017), and is present in other genera of the family with slight modifications occurring in the post-dehiscence processes of dispersal and seed burial (Yeo, 1984). In the *Pelargonium* genus, the structure of the awn is thinner and lighter and covered with feather-like hairs compared to the *Erodium* genus. This difference was explained as a condition necessary for the detachment from the *columella* through wind dispersal instead of being sprung away as *Erodium* seeds (Abraham and Elbaum, 2013). However, similarly to *Erodium*, once on the ground the awns start to coil in response to humidity, suggesting that the microstructure composing the awn especially, has evolved to function for self-burial only (Jung et al., 2014).

A focus on the awns coiling behavior has led to studies on the morphology and internal structure of Geraniaceae seeds, especially of *Erodium* and *Pelargonium* genus. In *Erodium*, the coiling movement was attributed to specialized cells containing tight helices of cellulose microfibrils, with an orientation almost parallel to the cell axis, rather than a bilayer effect (Abraham and Elbaum, 2013). Furthermore, a difference in the composition and distribution of materials along the awn was observed, with a higher concentration of modified lignin on the top part of the awn and a lower concentration of modified lignin in the lower part, which increases the hydrophobicity of the cell walls (Abraham et al., 2018). Differently in *Pelargonium* seeds, the awns are composed of a bi-layer structure with the hygroscopically active cells aligned along a layer of inactive cells (Jung et al., 2014; Shin et al., 2018).

In both cases, the driver for hygroscopic tissue expansion is determined by the arrangement of microfibrils forming the cell walls, composed of glycoproteins and cellulose, attached in multiple layers with different orientations (Jung et al., 2014).

The head part of Geraniaceae seeds, called capsule, plays a fundamental role in the anchoring of the seeds into the soil surface irregularities, such as crevices, which are then used by the seed to penetrate into it (Stamp, 1984; Evangelista et al., 2011). For instance, the capsules of *Erodium cicutarium* seeds showed a hooked, tapered carpel-tip which are fully covered from stiff directional hairs (Stamp, 1984). *Erodium* capsules were structurally modified (e g., hairs were removed with sandpaper, or tips were cut off) to investigate how the presence of hairs or tips affect the establishment of the seeds into soil substrates with different crevices sizes. The depths of burial, which indicates the height of the seed capsule above the substrates, were measured in both unmodified and modified seeds and in small or large crevices, showing that depths of *Erodium* seeds with unmodified capsules were greater in small crevices (Stamp, 1984). By using Scanning Electron Microscopy (SEM) investigations, directional hairs-like microstructures were found also in other plant families (eg., Poaceae), where they may help the seeds to move as a ratchet into the soil (Elbaum et al., 2007).

### Biomechanics

In self-burying seeds (e.g., *Erodium* and *Pelargonium* seeds) the motion is generated in a form of hygromorphic actuation, which is driven by the hygroscopic material - cellulose–that is a component of the awn fibrils (Evangelista et al., 2011). Cellulose absorbs water molecules, via hydrogen bonding, causing fibril volume expansion and swelling of wet tissues. Since water molecules adsorption in cellulose is reversible, the seed’s swollen tissue can shrink back to its original shape when dry (Jung et al., 2014). The amount of strain due to the variation of relative humidity (i.e., water adsorption) is called the Coefficient of Hygroscopic Expansion (CHE) (Dawson et al., 1997). To the best of our knowledge, the CHE was measured only for *Pelargonium appendiculatum* using a thermo-hygrostat (Ha et al., 2020). The measured value was equal to the measured one by Dawson et al., 1997 which studied the opening of the pinecones with a hygromorphic actuation mechanism similar to the Geraniaceae one. The value is reported in Table 1 and could be considered a suitable data for modelling the behavior of Geraniaceae in general.

**TABLE 1:** Summary of self-burying seeds biological features, measurements and characterization methods, and associated biological specifications for soft robotic solutions.

Self-burying seed		Morphometric and structural characteristics parameters		Biomechanics	
***Erodium*** 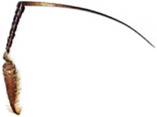 ***Pelargonium*** 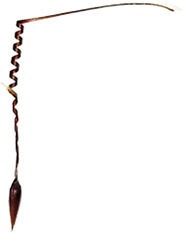	**Awn**	**Methodology**	**Biological specification**	**Methodology**	**Biological specification**
		• Morphometric analysis, sectioning, and weight measurements	• The driver for hygroscopic tissue expansion is determined by the arrangement of microfibrils forming the cell walls, composed of glycoproteins and cellulose, attached in multiple layers with different orientations (Jung et al., 2014) *Erodium cicutarium* • Maximum awn diameter: 0.002 ± 0.001 m • Awn height: 0.015 ± 0.005 m • Number of turns: 9 ± 2 • Awn spiral angle: 86 ± 2° • Section width: 0.001 m • Section height: 0.00025 m • Mass: 5 ± 1×10–6 kg (Evangelista et al., 2011) • Lignin composition and distribution (Abraham et al., 2018); • Arrangement of active and inactive layer (Abraham & Elbaum, 2013; Shin et al., 2018)	• Coefficient of Hygroscopic Expansion (CHE) measurement through mass changes in controlled relative humidity changes (Dawson et al., 1997) • Measurements of CHE with thermo-hygrostat (Ha et al., 2020)	Pinecones
					• CHE: 0.20 ± 0.04 for ΔRH = 1% (Dawson et al., 1997)
					*Pelargonium appendiculatum*
					• CHE: 0.15–0.2 (Ha et al., 2020)
				• Measurements of the Tilt angles (Ψ) and cellulose microfibril angle (MFAH) with Small Angle X-ray Scattering (SAXS) 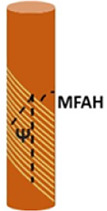	*Pelagonium peltatum*
					• *Ψ* = 5°, >3° and 19°
					• MAFH = 40, 16 and 70°
					*Erodium gruinum*
					• *Ψ* = 3°, >3° and 20°
					• MAFH = 30, 10 and 80°
					The three value are respectively for outermost sublayer, median sublayer and inner layer. (Abraham and Elbaum, 2013)
				• Measurements of helix Radios (R) and Pitch (P) using thermo-hygrostat 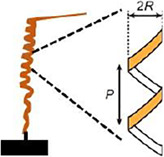	*Pelargonium appendiculatum*
					• R = 0.90 ± 0.43 mm
					• *p* = 1.85 ± 0.24 mm
					For RH 50%
					Both values increase with the increase of humidity (Ha et al., 2020)
				• Measurement of the Extensional force (E_F_) by constraining the increase in length with a loadcell 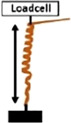 . • Measurement of Torque (T) by obstructing the rotation of the awn with a loadcell 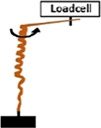	*Pelargonium carnosum*
					• E_F_ = 3.2 mN
					• T = 20 μN×m (moment arm: 20 mm)

				• Material biomechanical properties of *Erodium cicutarium* are assumed equal to the properties of wood (Ashby and Jones, 1996; Evangelista et al., 2011)	• Young’s modulus = 9 × 10^9^ Pa
					• Poisson’s ratio = 0.33
					• Shear modulus = 3.4 × 10 Pa (Ashby and Jones, 1996; Evangelista et al., 2011)
	**Capsule**	• Scanning electron microscopy (SEM)	The presence of hairs or tips affect the establishment of the seeds into soil substrates with different crevices sizes (Stamp, 1984)	• Drag force tests with load cell and dedicated setup	• F_drag_ _in beads_ = 2.5–3 mN (Jung et al., 2017)
		• Spraying with water to characterize the number of wet-dry cycles necessary for self-burial of seeds			
		• Observations of seed establishment of unmodified and modified seeds in different substrates (e. g. small or large crevices sizes)			
		• Measurement of the depth of burial of unmodified and modified seeds			

The typical helical configuration of the Gerianaceae awn is defined by two angles: the tilt angle (*Ψ*), which is the angle between the cellulose helix axis and the cell’s long axis; and the cellulose microfibril angle (MFAH) in relation to the cellulose helix axis and the cell’s long axis (Abraham and Elbaum, 2013). The *Ψ* and MFAH values change along the awn section of Gerianaceae and generate different expansion effects of bending and torsion of the awn. Values were measured for five Gerianaceae seeds via small angle X-ray scattering (SAXS) in different regions of the awn section.

The helical awn movement of Gerianaceae can be geometrically described by measuring the pitch (P) and radius (R) values of the helix during relative humidity changes (Ha et al., 2020).

Coiling and uncoiling of the awns of *Erodium* and ***Pelargonium*** seeds result in rotation and consequent digging of the seed in the soil fractures. Both movements are promoted by different biomechanical effects: a force due to coil expansion and torque caused by awn-tail rotation. Jung et al., 2014 measured, with a load cell, the extensional force of *Pelargonium carnosum* by constraining the increase in length with the increase of humidity. The authors also measured the torque by obstructing, with a load cell, the awn rotation during wetting. The maximum force was ∼1 mN and the torque 20 μN×m (moment arm = 20 mm). The authors proved that the torque decreased in magnitude over time, consistently with the decrease of Young’s modulus with increasing water content in the awn’s tissues (Jung et al., 2014).

To the best of our knowledge, Young’s modulus of the different sections of Geraniaceae awn seed has not been measured so far. However, Evangelista et al., 2011 took from the scientific literature the following values for the modelling of *Erodium cicutarium* trajectories: Young’s modulus = 9 × 109 Pa; Poisson’s ratio = 0.33, shear modulus = 3.4 × 10 Pa (Ashby and Jones, 1996). The value of Young’s modulus E was measured also in the wild wheat awns (Elbaum et al., 2007), finding values that range from 10 ± 2.8 GPa at the ridge, to 20.5 ± 2.6 GPa at the cap, where lignin is more abundant.

In relation to the seed capsule, few studies have focused on the measure of drag forces of three *Pelargonium* species (e g., *P. appendiculatum*, *P. carnosum* and *P. vitifolium*), which seem to strongly depend on seed capsules shape and size, as well as seed rotation (Jung et al., 2014; Jung et al., 2017). These experiments were typically performed using a dedicated setup with a load cell that pulled the seed capsules against a container filled with glass beads (i., e to mimic the soil environment), with or without rotation.

## Flying Seeds

Flying seeds can be divided into two major groups depending on their flight mechanism: the winged seeds and parachute seeds (Lee et al., 2014). The first type uses a gliding or autorotation thanks to a winged structure (e.g., *Acer* seed); the second one maximizes the drag force with hairy plumes (e.g., *Taraxacum* and *Tragopogon* seeds) (Minami and Azuma, 2003).

### Morphology and Structure

Winged seeds, or samaras, are dried fruits (achene) composed of a single fibrous wing, which wraps the seed (nut or pericarp). They can be found in different conditions such as tropical, temperate and alpine ecosystems. More than 140 genera, from 45 families and 25 orders have been identified as samara-bearing species, including taxa from species such as *Ailanthus altissima* (Mill.) Swingle, to common genera such as *Pinus*, *Fraxinus* and *Acer* (Manchester and O’Leary, 2010; Der Weduwen and Ruxton, 2019; Nave et al., 2021).

Although the similarity in the general structure, they can develop from either the style or the ovary wall (Mirle & Burnham, 1999) and the number of wings formed depends on the number of carpels composing the flowers. Furthermore, the venation pattern of the wings varies according to the tissue from which it is formed (Manchester and O’Leary, 2010). According to their symmetry, they can be divided into rolling and non-rolling samaras. Rolling samaras (e.g., *Fraxinus* spp.), which are symmetrical, both autogyrate and autorotate around their long-axis, whereas non-rolling samaras (e.g., *Acer* spp.), which are asymmetrical, only autogyrate downward along the vertical axis. This difference affects their descent speed and, as a consequence, their dispersal (Augspurger, 1986; Vogel, 2013).

Parachute seeds can be found in Compositae, Poaceae, Apocynaceae, and Salicaceae families, however, they exhibit different structures known as dandelion (Compositae), thistle (Compositae), eulalia (Poaceae), vine (Apocynaceae), and poplar (Salicaceae). They are characterized by fibrous structures, called pappi, which extend in a semi-sphere or circular cone serving as a parachute. In some cases, the pappi can have small protuberances, called spikes, or fine long appendages to increase the drag force during flight (Minami and Azuma, 2003). In the well-known dandelion seeds, e.g., *Taraxacum* and *Tragopogon* herbaceous species of the Compositae family, the pappus fibrous are connected to the achene, i.e. the single-seeded fruit, by a beak (Andersen, 1993). These structures, pappus and beak, besides facilitating flight, also facilitate germination by collecting rain droplets into the achene (Hale et al., 2010).

### Aerodynamics

#### Winged Seeds

Winged seed autorotation results from a delicate equilibrium between gravity (weight of the seed, center of mass) and inertia, as well as aerodynamic forces (Lee et al., 2014). Relevant major values for winged seeds characterization are: the center of mass (C_m_), which can be estimated by measuring the linear density along the seed axis through seed sectioning and weighting; and the aerodynamic parameters of descent speed (v_d_), rotational velocity (Ω), wing tip speed (v_t_), wing loading (W/S), coning angle (β) and windage coefficient (C_w_), which are used to describe the autorotation movement (Nave et al., 2021).

#### Parachute Seeds

In parachute seeds, key parameters for their dispersal performance include the horizontal dispersal distance, which is inversely proportional to their terminal velocity during vertical falling (*v*_*f*_) (Greene and Johnson, 1990), and changes with the seed mass (m) and the pappus projected area (A). Measurements of these parameters were reported for a *Taraxacum officinale* seed, with a *m* = 0.62 mg has a *v*_*f*_ = 0.27 m/s, and for a *Tragopogon pratensis* seed with *m* = 11.2 mg has a *v*_*f*_ = 0.57 m/s (Casseau et al., 2015). The *v*_*f*_ is conventionally calculated by chronometry, establishing the height from which the seed is dropped.

The laminar or turbulent nature of the flow moving over the plumes can be described using a non-dimensional aerodynamic parameter: the Reynolds number (*Re*) (Cummins et al., 2018). The flow through and around the pappus involves two different parameters, i.e., Re of the entire pappus and Re of an individual filament (Re_*f*_).

The drag coefficient (C_D_) is the other key aerodynamic parameter that is used to quantify the drag or resistance of the pappus seed in the air environment during the drop.

Recently Cummins et al., 2018 discovered that thanks to the pappus porosity two separated vortex rings appear onto the projected surface during the drop, stabilizing the vortex and maximizing the aerodynamic loading. The projected porosity is the ratio of the total projected area of the void spaces to the total plan area of the imaginary disk formed by the pappus and can be estimated with microscopy imaging and image processing (e.g., image binarization and pixels counting) (Casseau et al., 2015).

## From Biology to Engineering

The first biomechanical study of self-burying seed aimed at the fabrication of soft hygromorphic actuator was reported by Ha et al., 2020. The researchers constructed a theoretical model to explain the tissue-level behavior of the awns, comparing the results with the experimental measurements. The researchers fabricated an artificial hygromorphic actuator whose structure was bioinspired by the seed awns features (Pitch and Radius, MFAH and Ψ) to perform helical coiling. The hygro-responsive awn actuators were fabricated via the directional electrospinning process using PEO polymers (active and hygromorphic layer) onto a PI (polyimide) film of 50 µm (inactive layer) (Ha et al., 2020).

Artificial double-winged seeds were fabricated using 3D printing and characterized by Rabault et al., 2019 and Fauli et al., 2019. The experimental and theoretical models found an optimal wing fold angle able to minimize the descent velocity. Recently, artificial single-winged samaras bioinspired from *Acer platanoides* (Norway maple) seeds were 3D printed, using poly (lactic acid) (PLA). The researchers fabricated artificial seeds with similar morphological (shape, length, centers of mass and wing loading) and aerodynamic features (rotation velocities, wing tip speeds and lab descent speeds; Nave et al., 2021).

Artificial micro, meso and macro flying seeds (with half-widths of wings <1 mm, 1 mm and >1 mm, respectively) were fabricated with incorporated sensing and electronics (Kim et al., 2021). Micro-fabrication techniques were applied for the fabrication of fliers bioinspired to *Tristellateia* seeds using Silicon Nanomembranes (SiNMs) and organic polymers. In the study theoretical analysis and numerical simulation of the aerodynamics were carried out (Kim et al., 2021). The authors suggested the integration of the fliers with battery-free, wireless devices and colorimetric sensors for environmental purposes.

To explore the effects of pappus porosity, artificial silicon disks mimicking the pappus were microfabricated with a porosity of 92%, which is comparable to the one of a pappus (Cummins et al., 2018). The mean critical Re for *Taraxacum officinale* seed was 429, in good agreement with the critical Re value of 457 calculated for a silicon disk with the same porosity.

## Discussion

With a bioengineering approach, the study of biological models is necessary to identify and extract key features (in morphology, structure, biomechanics) that are relevant to the design and development of artificial systems (Margheri et al., 2012; Tramacere et al., 2013; Laschi and Mazzolai, 2016). The bioengineering approach has been used in plant-inspired robotics for investigating and then artificially translating several plant features (Mazzolai et al., 2020). With this review, authors aimed at the identification of the key parameters available at the state of the art in relation to the morphological computation abilities of self-burying and flying plant seeds which are relevant as insights for the design of artificial soft robotics solutions (Table 1 and 2).

**TABLE 2:** Summary of flying seeds biological features, measurements and characterization methods, and associated biological specifications for soft robotic solutions.

Flying seeds	Morphometric and structural characteristics parameters		Aerodynamics	
***Winged*** 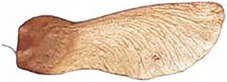	**Methodology**	**Biological specification**	**Methodology**	**Biological specification**
	• Morphometric analysis, sectioning, and weight measurements	*Acer platanoides*	• Seed sectioning and weighting for the centre of mass (Cm) measurement	*Acer platanoides*
		• Mass: 102.4 ± 30.8 mg		• Cm: 28.5 ± 4.0% of the seed’s length (Nave et al., 2021)
		• Length: 5.65 ± 0.76 cm (Nave et al., 2021)	• High frame rate camera picture and video for the measurement of descent speed (v_d_), rotational velocity (Ω), wing loading (W/S), windage coefficient (C_w_), and coning angle (β)	*Acer plantoides*
				• v_d_: 1.10 ± 0.24 m/s
				• Ω: 81.4 ± 27.6 rad/s
				• W/S: 2.09 ± 0.50 N/m^2^
				• Cw: 0.87 ± 0.39 (Nave et al., 2021)
				• β: 19.4 ± 2.5° (Lee et al., 2014)
***Parachutes*** 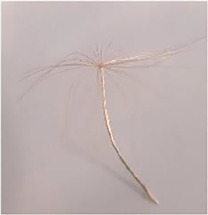	• Morphometric analysis, microscopy analysis, sectioning, and weight measurements	*Taraxacum officinale*	• Microscopy imaging and processing for the measurement of the projected surface area (A) and for the projected porosity (P)	*Taraxacum officinale*
		• Total mass: 0.633 mg	• High frame rate camera picture and video for the measurement of descent speed (U) and for the calculation of coefficient of Reynolds for pappus (Re) and single filament (R_ef_) and Drag Coefficient (C_D_)	• A *=* 12.6 mm^2^
		• Number of ribs: 100		• *p* = 91.6%
		• Main rib length: 7.4 mm		• U = 0.39 m/s
		• Main rib mean diameter: 16.3 μm (Cummins et al., 2018)		• R_e_ = 357
		*Tragopogon pratensis*		• R_ef_ = 0.422
		• Parachute mass: 2.03 ± 0.31 mg		• C_D_∼ 5 (Cummins et al., 2018)
		• Total mass:11.32 ± 0.27		*Tragopogon pratensis*
		• % mass allocated in the parachute:18%		• *p* = 90.5%
		• Main rib length: 18.43 ± 0.72 mm		• U = 0.34–0.57 m/s
		• Number of ribs: 27.6 ± 0.60		• CD = 1.15–1.26 (Casseau et al., 2015)
		• Rib inclination in the horizontal plane: 21.01 ± 2.28°		
		• Main rib mean diameter: 91.80 ± 18.62 μm (Casseau et al., 2015)		

Additional focused studies of the morphology and internal structure and composition of the seeds, and the associated biomechanics and aerodynamics behavior in different environmental conditions, would be relevant to further explore the potential use of seed-inspired solutions for soft robotics.

The outcomes obtained by this approach, provide a mutual biology-robotics benefit: they are a scientific result for plant biology, by providing new knowledge on plant seeds characteristics and on the associated dispersal strategy; and at the same time they represent a series of innovation guidelines for the design of artificial multi-functional materials, soft miniaturized robots and the associated morphological computation behaviors.

## References

[B1] Abraham Y. Dong Y. Aharoni A. Elbaum R. (2018). Mapping of Cell wall Aromatic Moieties and Their Effect on Hygroscopic Movement in the Awns of Stork's Bill. Cellulose 25 (7), 3827–3841. 10.1007/s10570-018-1852-x

[B2] Abraham Y. Elbaum R. (2013). Hygroscopic Movements in Geraniaceae: the Structural Variations that Are Responsible for Coiling or Bending. New Phytol. 199 (2), 584–594. 10.1111/nph.12254 23574364

[B4] Andersen M. C. (1993). Diaspore Morphology and Seed Dispersal in Several Wind-Dispersed Asteraceae. Am. J. Bot. 80, 487–492. 10.1002/j.1537-2197.1993.tb13830.x 30139156

[B5] Armon S. Efrati E. Kupferman R. Sharon E. (2011). Geometry and Mechanics in the Opening of Chiral Seed Pods. Science 333 (6050), 1726–1730. 10.1126/science.1203874 21940888

[B6] Ashby M. F. Jones D. (1996). Engineering Materials I. 2nd edition. Oxford: Butterworth-Heinemann.

[B7] Augspurger C. K. (1986). Morphology and Dispersal Potential of Wind‐dispersed Diaspores of Neotropical Trees. Am. J. Bot. 73 (3), 353–363. 10.1002/j.1537-2197.1986.tb12048.x

[B8] Casseau V. De Croon G. Izzo D. Pandolfi C. (2015). Morphologic and Aerodynamic Considerations Regarding the Plumed Seeds of Tragopogon Pratensis and Their Implications for Seed Dispersal. PLoS One 10, e0125040. 10.1371/journal.pone.0125040 25938765 PMC4418730

[B9] Cummins C. Seale M. Macente A. Certini D. Mastropaolo E. Viola I. M. (2018). A Separated Vortex Ring Underlies the Flight of the Dandelion. Nature 562 (7727), 414–418. 10.1038/s41586-018-0604-2 30333579

[B10] Dawson C. Vincent J. F. V. Rocca A.-M. (1997). How pine Cones Open. Nature 390, 668. 10.1038/37745

[B11] De Mestral G. (1961). Separable Fastening Device. U.S. Patent No. 3,009,235. Washington, DC: U.S. Patent and Trademark Office.

[B12] Elbaum R. Zaltzman L. Burgert I. Fratzl P. (2007). The Role of Wheat Awns in the Seed Dispersal Unit. Science 316 (5826), 884–886. 10.1126/science.1140097 17495170

[B13] Evangelista D. Hotton S. Dumais J. (2011). The Mechanics of Explosive Dispersal and Self-Burial in the Seeds of the Filaree, Erodium Cicutarium (Geraniaceae). J. Exp. Biol. 214 (4), 521–529. 10.1242/jeb.050567 21270299

[B14] Fauli R. A. Rabault J. Carlson A. (2019). Effect of wing Fold Angles on the Terminal Descent Velocity of Double-Winged Autorotating Seeds, Fruits, and Other Diaspores. Phys. Rev. E 100, 13108. 10.1103/physreve.100.013108 31499848

[B15] Fratzl P. Barth F. G. (2009). Biomaterial Systems for Mechanosensing and Actuation. Nature 462 (7272), 442–448. 10.1038/nature08603 19940914

[B16] Geer R. Iannucci S. Li S. (2020). Pneumatic Coiling Actuator Inspired by the Awns of *Erodium Cicutarium* . Front. Robot. AI 7, 17. 10.3389/frobt.2020.00017 33501186 PMC7805895

[B17] Greene D. F. Johnson E. A. (1990). The Aerodynamics of Plumed Seeds. Funct. Ecol. 4, 117–125. 10.2307/2389661

[B18] Ha J. Choi S. M. Shin B. Lee M. Jung W. Kim H.-Y. (2020). Hygroresponsive Coiling of Seed Awns and Soft Actuators. Extreme Mech. Lett. 38, 100746. 10.1016/j.eml.2020.100746

[B19] Hale A. N. Imfeld S. M. Hart C. E. Gribbins K. M. Yoder J. A. Collier M. H. (2010). Weed Sci. 58, 4. 10.1614/ws-d-10-00036.1

[B20] Hutchinson J. (1969). Evolution and Phylogeny of Flowering Plants. London: Academic Press, 717.

[B21] Johnson J. S. Cantrell R. S. Cosner C. Hartig F. Hastings A. Rogers H. S. (2019). Rapid Changes in Seed Dispersal Traits May Modify Plant Responses to Global Change. AoB Plants 11 (3), plz020. 10.1093/aobpla/plz020 31198528 PMC6548345

[B22] Jung W. Choi S. M. Kim W. Kim H.-Y. (2017). Reduction of Granular Drag Inspired by Self-Burrowing Rotary Seeds. Phys. Fluids 29 (4), 041702. 10.1063/1.4979998

[B23] Jung W. Kim W. Kim H.-Y. (2014). Self-burial Mechanics of Hygroscopically Responsive Awns. Integr. Comp. Biol. 54 (6), 1034–1042. 10.1093/icb/icu026 24760793

[B24] Kim B. H. Li K. Kim J.-T. Park Y. Jang H. Wang X. (2021). Three-dimensional Electronic Microfliers Inspired by Wind-Dispersed Seeds. Nature 597, 503–510. 10.1038/s41586-021-03847-y 34552257

[B25] Laschi C. Mazzolai B. (2016). Lessons from Animals and Plants: The Symbiosis of Morphological Computation and Soft Robotics. IEEE Robot. Automat. Mag. 23 (3), 107–114. 10.1109/mra.2016.2582726

[B26] Lee S. J. Lee E. J. Sohn M. H. (2014). Mechanism of Autorotation Flight of maple Samaras (Acer Palmatum). Exp. Fluids 55 (4), 1718. 10.1007/s00348-014-1718-4

[B27] Manchester S. R. O’Leary E. L. (2010). Phylogenetic Distribution and Identification of Fin-Winged Fruits. Bot. Rev. 76 (1), 1–82. 10.1007/s12229-010-9041-0

[B28] Marcussen T. Meseguer A. S. (2017). Species-level Phylogeny, Fruit Evolution and Diversification History of Geranium (Geraniaceae). Mol. Phylogenet. Evol. 110, 134–149. 10.1016/j.ympev.2017.03.012 28288945

[B29] Margheri L. Laschi C. Mazzolai B. (2012). Soft Robotic Arm Inspired by the octopus: I. From Biological Functions to Artificial Requirements. Bioinspir. Biomim. 7 (2), 025004. 10.1088/1748-3182/7/2/025004 22617132

[B30] Mazzolai B. Tramacere F. Fiorello I. Margheri L. (2020). The Bio-Engineering Approach for Plant Investigations and Growing Robots. A Mini-Review. Front. Robot AI 7, 573014. 10.3389/frobt.2020.573014 33501333 PMC7806088

[B31] Minami S. Azuma A. (2003). Various Flying Modes of Wind-Dispersal Seeds. J. Theor. Biol. 225 (1), 1–14. 10.1016/s0022-5193(03)00216-9 14559055

[B32] Mirle C. Burnham R. J. (1999). Identification of Asymmetrically Winged Samaras from the Western Hemisphere. Brittonia 51 (1), 1–14. 10.2307/2666549

[B33] Nave G. K. Hall N. Somers K. Davis B. Gruszewski H. Powers C. (2021). Wind Dispersal of Natural and Biomimetic Maple Samaras. Biomimetics 6 (2), 23. 10.3390/biomimetics6020023 33805294 PMC8103264

[B34] Pandolfi C. Izzo D. (2013). Biomimetics on Seed Dispersal: Survey and Insights for Space Exploration. Bioinspir. Biomim. 8 (2), 025003. 10.1088/1748-3182/8/2/025003 23648867

[B35] Rabault J. Fauli R. A. Carlson A. (2019). Curving to Fly: Synthetic Adaptation Unveils Optimal Flight Performance of Whirling Fruits. Phys. Rev. Lett. 122, 24501. 10.1103/physrevlett.122.024501 30720318

[B36] Shin B. Ha J. Lee M. Park K. Park G. H. Choi T. H. (2018). Hygrobot: A Self-Locomotive Ratcheted Actuator Powered by Environmental Humidity. Sci. Robot 3 (14), eaar2629. 10.1126/scirobotics.aar2629 33141700

[B37] Stamp N. E. (1984). Self-burial Behaviour of Erodium Cicutarium Seeds. J. Ecol. 72, 611–620. 10.2307/2260070

[B38] The Plant List (2013). “The Plant List,”. Version 1.1. Available at: http://www.theplantlist.org/ (accessed January 1, 2013).

[B39] Tramacere F. Beccai L. Kuba M. Gozzi A. Bifone A. Mazzolai B. (2013). The Morphology and Adhesion Mechanism of *Octopus vulgaris* Suckers. PLoS One 8 (6), e65074. 10.1371/journal.pone.0065074 23750233 PMC3672162

[B40] Traveset A. Heleno R. Nogales M. (2014). The Ecology of Seed Dispersal. Seeds: Ecol. Regen. Plant communities 3, 62–93. 10.1079/9781780641836.0062

[B41] Vogel S. (2013). “About Lift,” in Comparative Biomechanics: Life’s Physical World. Second Edition (Princeton University Press), 225–249.

[B42] Weduwen D. Ruxton G. D. (2019). Secondary Dispersal Mechanisms of Winged Seeds: a Review. Biol. Rev. 94, 1830–1838. 10.1111/brv.12537 31215745

[B43] Yao L. Ishii H. (2019). “Hygromorphic Living Materials for Shape Changing,” in Robotic Systems and Autonomous Platforms. (Cambridge, United Kingdom: Woodhead Publishing), 41–57. 10.1016/b978-0-08-102260-3.00003-2

[B44] Yeo P. F. (1984). Fruit-discharge-type in Geranium (Geraniaceae): its Use in Classification and its Evolutionary Implications. Bot. J. Linn. Soc. 89, 1–36. 10.1111/j.1095-8339.1984.tb00998.x

